# Cost-effectiveness of telehealth for patients with depression: evidence from the Healthlines randomised controlled trial

**DOI:** 10.1192/bjpo.bp.116.002907

**Published:** 2016-08-09

**Authors:** Padraig Dixon, Sandra Hollinghurst, Louisa Edwards, Clare Thomas, Alexis Foster, Ben Davies, Daisy Gaunt, Alan A. Montgomery, Chris Salisbury

**Affiliations:** **Padraig Dixon,** DPhil, Centre for Academic Primary Care, School of Social and Community Medicine, University of Bristol, Bristol, UK; **Sandra Hollinghurst,** PhD, Centre for Academic Primary Care, School of Social and Community Medicine, University of Bristol, Bristol, UK; **Louisa Edwards,** PhD, Centre for Academic Primary Care, School of Social and Community Medicine, University of Bristol, Bristol, UK; **Clare Thomas,** PhD, Centre for Academic Primary Care, School of Social and Community Medicine, University of Bristol, Bristol, UK; **Alexis Foster,** MPH, Clinical Trials Research Unit, School of Health and Related Research (ScHARR), University of Sheffield, Sheffield, UK; **Ben Davies,** PhD, Centre for Academic Primary Care, School of Social and Community Medicine, University of Bristol, Bristol, UK; **Daisy Gaunt,** MSc, Bristol Randomised Trials Collaboration (BRTC), School of Social and Community Medicine, University of Bristol, Bristol, UK; **Alan A. Montgomery,** PhD, Bristol Randomised Trials Collaboration (BRTC), School of Social and Community Medicine, University of Bristol, Bristol, UK; Nottingham Clinical Trials Unit, Faculty of Medicine & Health Sciences, Queen’s Medical Centre, University of Nottingham, Nottingham, UK; **Chris Salisbury,** MD, Centre for Academic Primary Care, School of Social and Community Medicine, University of Bristol, Bristol, UK

## Abstract

**Background:**

Depression is a prevalent long-term condition that is associated with substantial resource use. Telehealth may offer a cost-effective means of supporting the management of people with depression.

**Aims:**

To investigate the cost-effectiveness of a telehealth intervention (‘Healthlines') for patients with depression.

**Method:**

A prospective patient-level economic evaluation conducted alongside a randomised controlled trial. Patients were recruited through primary care, and the intervention was delivered via a telehealth service. Participants with a confirmed diagnosis of depression and PHQ-9 score ≥10 were recruited from 43 English general practices. A series of up to 10 scripted, theory-led, telephone encounters with health information advisers supported participants to effect a behaviour change, use online resources, optimise medication and improve adherence. The intervention was delivered alongside usual care and was designed to support rather than duplicate primary care. Cost-effectiveness from a combined health and social care perspective was measured by net monetary benefit at the end of 12 months of follow-up, calculated from incremental cost and incremental quality-adjusted life years (QALYs). Cost–consequence analysis included cost of lost productivity, participant out-of-pocket expenditure and the clinical outcome.

**Results:**

A total of 609 participants were randomised – 307 to receive the Healthlines intervention plus usual care and 302 to receive usual care alone. Forty-five per cent of participants had missing quality of life data, 41% had missing cost data and 51% of participants had missing data on either cost or utility, or both. Multiple imputation was used for the base-case analysis. The intervention was associated with incremental mean per-patient National Health Service/personal social services cost of £168 (95% CI £43 to £294) and an incremental QALY gain of 0.001 (95% CI −0.023 to 0.026). The incremental cost-effectiveness ratio was £132 630. Net monetary benefit at a cost-effectiveness threshold of £20 000 was −£143 (95% CI −£164 to −£122) and the probability of the intervention being cost-effective at this threshold value was 0.30. Productivity costs were higher in the intervention arm, but out-of-pocket expenses were lower.

**Conclusions:**

The Healthlines service was acceptable to patients as a means of condition management, and response to treatment after 4 months was higher for participants randomised to the intervention. However, the positive average intervention effect size was modest, and incremental costs were high relative to a small incremental QALY gain at 12 months. The intervention is not likely to be cost-effective in its current form.

**Declaration of interest:**

None.

**Copyright and usage:**

© The Royal College of Psychiatrists 2016. This is an open access article distributed under the terms of the Creative Commons Attribution (CC BY) licence.

The management of long-term conditions is challenging. Some 30% of the population of England are estimated to have a long-term condition,^1^ whereas their treatment constitutes 70% of all health and care expenditure in England.^1^ Depression may be considered as an exemplar long-term condition: it is prevalent,^2^ recurrent,^3^ associated with morbidity and comorbidity,^4^ mortality^4^ and substantial resource use.^5^

One form of care that may be relevant to the management of depression (and other long-term conditions) is telehealth.^6^ Telehealth can be defined as the use of technology to deliver healthcare remotely and to promote patient self-management.^7^ A multicentre, parallel, two-arm, individually randomised controlled trial (RCT) was undertaken to assess the effectiveness and cost-effectiveness of a structured, theory-driven telehealth intervention – ‘Healthlines' – for patients with depression. This paper describes the results of an economic evaluation conducted alongside the RCT in order to estimate the cost-effectiveness of the Healthlines service for primary care patients with depression.

## Method

Patients with a confirmed diagnosis of depression, and a score ≥10 on the validated^8^ and widely used^9^ nine-item Patient Health Questionnaire (PHQ-9) depression scale^10^ were recruited from 43 general practices in or near the English cities of Bristol, Sheffield and Southampton. The design of the telehealth intervention,^11^ the protocol for the RCT^12^ and results of the RCT^13^^,^^14^ have been reported elsewhere. In brief, 609 patients were individually randomised to receive either usual care or usual care plus the Healthlines service. Patients were followed up for 12 months.

The main perspective adopted for the economic evaluation, in line with National Institute for Health and Care Excellence (NICE) guidance,^15^ was that of the health and social care provider. A cost-effectiveness analysis was carried out from this perspective comparing costs, to the UK National Health Service (NHS) and personal social services (PSS) sectors, with quality-adjusted life years (QALYs). We also separately report the impact of the intervention on personal expenditure and on productivity as part of a cost–consequence analysis.

### Intervention

The Healthlines service was developed based on a programme of work^13^ that encompassed a systematic review of effective telehealth interventions,^16^ qualitative work on patient and provider experiences of telehealth,^17^ a survey of patient attitudes toward telehealth^18^ and reviews of evidence-based treatment guidelines for depression. This work informed the development and delivery of the *de novo* telehealth intervention^11^ delivered in the RCT.

The intervention consisted of telephone support designed to encourage participants to use resources available from the internet, along with efforts to optimise treatment and promote medication adherence. Resources available included a self-directed cognitive–behavioural therapy (CBT) course involving the use of a book or the CBT website ‘Living Life to the Full Interactive’ (LLTTFi); access to a secure web portal which included health information; access to the Big White Wall (BWW) online forum; requests for discussions with the trial's trained health information advisors (HIAs – see below); and copies of letters sent to the participant's general practitioner (GP).

Scripted, scheduled telephone encounters took place over the 12 months of trial follow-up between trial participants and HIAs. The HIAs, non-clinical staff with experience in providing health information to members of the public by telephone, were provided with additional training in order to deliver the Healthlines service. They were employed by the NHS, and they received supervision from nursing staff and from pharmacists. Participants randomised to the intervention were eligible to receive a maximum of 10 such telephone-based encounters and also receive usual care.

Participants randomised to the control arm received usual care for depression.

### Measurement of outcomes

The primary clinical outcome for the trial was the proportion of patients responding to treatment at 4 months from randomisation, measured using the PHQ-9 questionnaire. Response was defined as a reduction (from baseline) in PHQ-9 of at least 5 points and an overall score of less than 10. PHQ-9 responses were also collected at 8 and 12 months.

Information on health-related quality of life was collected at baseline, 4, 8 and 12 months using the EQ-5D-5L questionnaire,^19^ a standardised, generic instrument that allows for the measurement and valuation of health-related quality of life. The EQ-5D-5L measures five dimensions of health-related quality of life (mobility, self-care, usual activities, pain and discomfort, and anxiety and depression) with five categories of health states corresponding to no problems, slight problems, moderate problems, severe problems and extreme problems. The EQ-5D-3L version of the questionnaire is known to discriminate between different levels of depression severity^20^ and has ‘adequate' validity and responsiveness in patients with depression.^21^

### Measurement and valuation of resource use

With participant consent, details of all primary care consultations and relevant prescriptions (antidepressants, hypnotics and anxiolytics, and drugs used in psychoses and related disorders) issued during the 12-month follow-up period were extracted from GP records.

Questionnaires administered at 4, 8 and 12 months requested information on other healthcare and social care accessed for reasons connected with the participant's depression. This questionnaire, versions of which have been used in previous within-trial primary care evaluations, is available from the ‘Database of Instruments for Resource Use Measurement' at www.dirum.org. The questionnaire included the use of hospital services, attendance at accident and emergency departments, ambulance use, and the use of PSS such as social worker services. The questionnaires also requested information on the use of private therapies, out-of-pocket expenditure, time-off from work, benefits received, informal care and the use of voluntary-sector services. The questionnaires could be completed online or by post, but those completing them online still had to complete the EQ-5D by post because of licencing restrictions.

All resources were valued and costs reported in pound sterling at 2012/13 prices. Curtis^22^ was used to cost primary care consultations, community health and social care where possible. NHS National Reference Costs for 2012/13^23^ were used to value hospital and ambulance use. All sources are described in the supplementary material (see in particular Tables DS1 and DS2). The costs of drugs prescribed in primary care and reported in medical records were based on the Prescription Cost Analysis England database^24^ and cross-checked against the British National Formulary. The value of personal expenditure was obtained directly from participant responses to questionnaires.

Productivity costs were calculated from reports of working days missed by trial participants, by friends and relatives as a consequence of the participant's depression, and time from work to attend healthcare appointments. All of this time was valued at the national median gross hourly wage of £11.59 for 2013.^25^

Data on the resources used to provide the Healthlines service were extracted from provider computer systems. This included the number and length of all telephone calls, the number of attempts by a HIA to contact a participant, the use of the LLTTFi website, the number of people who were sent the designated CBT book, and the use of the BWW website. Resources involved in establishing the service included training costs and the purchase of licences for LLTTFi and BWW, and these were included in the calculation of intervention cost. Table DS3 in the online supplementary material summarises the unit costs of these resources.

The HIAs were remunerated at Band 4 of the NHS ‘Agenda for Change' pay scale. Cost-per-hour was estimated using the framework of Curtis,^22^ adjusted to reflect the working pattern of the HIAs. Anonymised task-scheduling diaries kept by the HIAs were used to estimate the proportion of their time spent not in contact with intervention participants: the ratio of contact/non-contact time was estimated to be 2:1. Further adjustment was made to allow for the 40-h workweek of the HIAs. HIAs received initial and ongoing training from a nurse-grade trainer and from a consultant psychiatrist in the LLTTFi package. The costs of training were amortised over an assumed duration of 3 years. This reflects the consideration that any training received would be relevant to the service for at least 3 years, after which additional training might become necessary. We regard this as a conservative assumption.

### Analysis of data

All analyses were conducted in Stata 13.1 (Statacorp: College Station, Texas).

The distribution of all data used in the analysis was inspected and summary measures such as means and standard deviations calculated. The data were inspected for missingness. Data relating to the use of the intervention and primary care data (including prescriptions) were generally complete. Less than 0.5% of data relating to primary care consultations were missing. These missing values were imputed with mean values at the participant level. The resulting data-set formed our ‘available cases'. The predominant sources of any remaining missing data were questionnaire responses, particularly questions relating to resource use and quality of life. In spite of satisfactory data completeness on primary care and the intervention, missing questionnaire responses meant that 45% of participants had missing quality of life data, 41% had missing cost data, and 51% of participants had missing data on either cost data or utility data, or both. Complete cases had complete data on all cost items and all quality of life data items at all time points, and 49% of participants (47% in the intervention arm, 51% in the control arm) qualified as complete cases under this definition.

Missing data were imputed using multiple imputation by chained equations, as implemented by the – ice – command^26^^,^^27^ in Stata 13.1. Data were assumed to be missing at random (MAR). The imputation model was stratified by trial arm^27^ and included demographic and cost variables without missing data alongside clinical outcome variables at baseline and follow-up, cost and utility variables with missing data, depression history, baseline depression status and whether patients were being prescribed antidepressants. Predictive mean matching was used to account for non-normal distributions in some included variables.^27^ Passive imputation was performed for categorical variables, such as binary PHQ-9 scores, that were functions of imputed variables.

Costs were imputed at the level of the major aggregate costs (e.g. primary care, medication, PSS costs and other NHS costs). The number of imputations (*n*=60) was selected to be greater than the proportion of missing data following White *et al*.^27^

Health-related quality of life utility measures at each follow-up time point were imputed, which were then used to generate QALY estimates. Utility values were calculated at baseline and the three follow-up time points. These data were obtained from participant responses to the EQ-5D-5L. We used the Euroqol UK crosswalk value set for mapping responses to the three-level version of EQ-5D.^28^ QALYs were calculated from the utility data using the ‘area under-the-curve' method and adjusted for baseline differences in EQ-5D-5L scores.^29^

Cost-effectiveness analysis was performed on the imputed dataset using the methods described in Faria *et al*,^30^ which implement ‘Rubin's rules',^31^ in order to reflect the variation within and between the 60 imputed data-sets. Regression analysis was used to generate the cost-effectiveness results and to characterise the uncertainty surrounding point estimates. Seemingly unrelated regression (SUR) was used to jointly model costs and QALYs using the – sureg – command in Stata. Baseline imbalances in utility were controlled for in the regression model.^32^

Incremental cost-effectiveness ratios (ICERs) were calculated from estimated coefficients. To avoid the complications^33^^,^^34^ of calculating confidence intervals around ICERs, confidence intervals are presented only for net benefit statistics, which were calculated parametrically from regression output.^35^ Net monetary benefit estimates were calculated using threshold values of £20 000 and £30 000 suggested by the NICE for use within NHS decision-making.^15^ Costs and outcomes were not discounted as trial follow-up was limited to 12 months. Analysis was conducted using an ‘intention to treat' approach.

### Sensitivity analysis

The base-case cost-effectiveness analysis used imputed data. As a sensitivity check, cost-effectiveness was also estimated on complete cases only and the results were compared. For both imputed and complete case data, probabilistic sensitivity analysis was implemented by calculating cost-effectiveness acceptability curves (CEACs) to quantify uncertainty around point estimates of net monetary benefit.

One-way sensitivity analysis was also performed. The sensitivity of the imputed results to self-reported use of secondary care was tested by removing these costs. Although all questionnaire responses concerning secondary care use were checked by a clinician for relevance to depression, the sensitivity analysis provides a means of assessing whether recall bias, misclassification or infrequent but expensive events differed between arms. The sensitivity of the results to the cost of an element of the intervention – that of the BWW licence – was also assessed in order to account for the possibility that these licence costs would be lower if the intervention were rolled out across the NHS.

## Results

A total of 307 participants were randomised to receive the intervention and 302 to the control arm. The mean age in the control (intervention) arm was 50.0 (49.1) years; 68% (69%) were female and 97% (98%) of participants were of White ethnicity. The mean PHQ-9 score at baseline was 16.7 (17.1), 93% (91%) of respondents had previously been treated for depression, and 90% (87%) of participants were taking antidepressants at the time of randomisation.

### Resource use and cost

Twenty-six per cent of participants, of those known to have received any calls, received none or little of the intervention (defined as starting two or fewer telephone encounters), 44% received some of the planned intervention sessions (3–8 encounters) and 29% of participants received all or almost all of the intervention (9–10 encounters). The median number of encounters was 5 (interquartile range: 2 to 9), and the mean duration of an encounter was 18.5 min.

The mean per participant cost of the intervention was £113, of which costs associated with telephone encounter calls (such as HIA remuneration and associated on-costs) comprised 66% of the total (Table 1).

Patients in the intervention arm incurred more primary care costs than did patients in the control arm, primarily because of a greater number of GP consultations. Prescription costs were similar between arms. Intervention patients incurred higher secondary care costs but lower PSS costs. We use imputed data for our base-case results and because we imputed at the level of aggregate cost categories rather than at the resource level, we present these costs in Table 2. Detailed, disaggregated resource use data on available and complete cases are presented in the supplementary material in Tables DS4 to DS10.

**Table 1:** Mean (s.d.) depression intervention cost (£) per participant for all participants and complete cases

Intervention elements	All intervention participants (*n*=308)^a^ mean £ (s.d.)	Complete cases (*n*=145)^a^ mean £ (s.d.)
Encounter calls	71.84 (57.64)	86.55 (56.39)
Non-scheduled calls	3.26 (7.17)	3.58 (6.51)
All calls	75.11 (61.04)	90.13 (59.18)
LLTTFi website	9.74 (6.5)	12.18 (6.39)
ODLM book	9.09 (19.98)	9.45 (12.53)
Big White Wall	19.09 (12.75)	23.88 (12.53)
Total cost per participant	113.03 (80.46)	135.68 (77.93)

LLTTFi, Living Life to the Full Interactive online cognitive-behavioural therapy programme; ODLM, *Overcoming Depression and Low Mood: A Five Areas Approach* cognitive–behavioural therapy book.^36^

a Includes one usual care participant who received the intervention in part in error.

**Table 2:** Imputed NHS and PSS costs

Imputed costs	*N*^a^	Usual care mean £ (s.e.)^b^	Intervention mean £ (s.e.)^b^
Imputed mean primary care costs	609	362 (15)	404 (17)
Imputed mean drug costs	609	88 (12)	92 (10)
Imputed mean hospital, ambulance and other non-primary care NHS costs	609	230 (34)	263 (37)
Imputed mean intervention cost	609	–	113 (5)
Imputed mean NHS costs, including the intervention	609	680 (41)	872 (46)
Imputed mean PSS costs	609	38 (13)	14 (5)
Imputed mean NHS and PSS costs, including the intervention	609	718 (45)	886 (47)

PSS, personal social services.

a This sample size is based on 60 imputed datasets.

b Standard errors – rather than standard deviations – are reported for imputed data.

### Quality of life

The QALYs were calculated to adjust for baseline EQ-5D scores (Table 3).The control arm baseline mean EQ-5D score was 0.52 (s.e.=0.02) compared with an intervention baseline EQ-5D score of 0.51 (s.e.=0.02), calculated using the imputed data-set.

**Table 3:** Imputed QALYs

	*N*^a^	Usual care mean (s.e.)	Intervention mean (s.e.)
Imputed adjusted QALYs	609	0.540 (0.009)	0.541 (0.009)

QALYs = quality-adjusted life years.

a This sample size is based on 60 imputed datasets.

### Cost-consequence results

Table 4 relates major cost categories to outcomes in a cost–consequence matrix, using available data (i.e. before multiple imputation of missing values).

**Table 4:** Cost-consequence matrix (based on available cases)

Available data on costs and consequences	Usual care	*N* (usual care)	Intervention	*N* (intervention)	Difference (95% CI)
Costs (£)					
Mean cost of intervention	0^‡^	188	136	169	–
Mean cost of NHS resources, excluding cost of intervention	645	188	709	169	64 (−76 to 193)^a^
Mean cost of NHS resources, including intervention	646	188	845	169	199 (79 to 339)^a^
Mean cost of PSS	37	193	15	171	−21 (−73 to 1)^a^
Mean cost of NHS and PSS resources, including intervention	683	188	860	169	177 (41 to 317)^a^
Out-of-pocket expenses	199	246	177	233	−21 (−116 to 57)^a^
Mean societal value per patient of lost production	74	246	242	233	168 (45 to 362)^a^
Consequences^b^					
PHQ-9 response at 4 months, adjusted for site and baseline PHQ-9	19%	270	27%	255	Odds ratio 1.7 (1.1 to 2.5)
Adjusted mean PHQ-9^c^	12.0	261	11.5	255	−0.5 (−1.5 to 0.5)
EQ-5D-5L^d^	0.564	227	0.569	219	0.005 (−0.053 to 0.061)^a^
Adjusted QALYs^d^	0.536	175	0.567	158	0.031 (−0.022 to 0.0810)^a^

CI, confidence interval; PSS, personal social services; PHQ-9, Patient Health Questionnaire; QALYs, quality-adjusted life years. Costs are reported accurate to £1 and may not sum to apparent totals owing to the effects of rounding.

‡ One participant in the control arm received elements of the intervention, the estimated cost of which was estimated to be £0.38.

a Confidence interval calculated as accelerated and bias corrected interval from 1000 bootstrap replicates to account for the skewed distribution of costs.

b Except where otherwise stated, all consequences were measured at 12 months, or over a period up to 12 months.

c Measured as the adjusted mean difference in continuous PHQ-9 scores at 12 months.

d Based on available data, and adjusted for baseline EQ-5D responses.

NHS/PSS costs were significantly higher in the intervention arm, largely owing to the cost of the intervention. Participants in the control arm reported higher expenditure on private healthcare costs such as private counselling, psychotherapy, psychiatry and complementary/alternative remedies (mean per patient in available cases: £47.54) than in the intervention arm (mean per patient in available cases: £39.28). Patients also reported slightly higher expenditure in the control arm compared with the intervention arm on out-of-pocket expenses connected with their condition such as self-help books and gym memberships.

The value of lost production was approximately three times higher in the intervention arm for the following reasons. Intervention participants reported a greater number of days affected by depression (mean per patient in available cases: 12.4 working days (s.d.=42.0) in the intervention arm compared with 5.2 working days (s.d.=15.7)) and more hours taken from work to attend healthcare appointments (mean per patient in available cases: 12.8 h (s.d.=86.8) in the intervention arm compared with 2.9 h (s.d.=12.5)).

These findings are influenced by a small number of individuals in the intervention arm who reported large amounts of lost time, and hence these results are probably owing to chance imbalance. No participant in the control arm reported more than 120 working days lost owing to depression, whereas 11 individuals in the intervention arm had at least 120 working days lost, with a mean value among this group of individuals of 187 days.

The consequences of the intervention include a significant increase in the proportion of responders to treatment, corresponding to a ‘number needed to treat' of 12, and a reduction in mean PHQ-9 scores of −0.5 (95% confidence interval −1.5 to 0.5).

### Cost-effectiveness analysis

Base-case cost-effectiveness results, using imputed data, from the perspective of a health and social care provider (i.e. NHS/PSS) are presented in Table 5. Results from an NHS-only perspective are presented in online supplementary material (Table DS11 and Fig. DS1) and are similar.

**Table 5:** Cost-effectiveness of the Healthlines intervention from an NHS/PSS perspective

	Usual care mean	Intervention mean	Difference (95% CI)
Costs and QALYs			
Total NHS and PSS costs	£718	£886	£168 (£43 to £294)
QALYs	0.540	0.541	0.001 (−0.023 to 0.026)
Cost-effectiveness statistics			
ICER: £132 630			
Probability that intervention cost-effective at CE threshold of £20 000: 0.30			
Probability that intervention cost-effective at CE threshold of £30 000: 0.37			
NMB at threshold of £20 000 (95% confidence interval): −£143 (−£164 to −122)			

CI, confidence interval; PSS, personal social services; QALYs, quality-adjusted life years; ICER, incremental cost-effectiveness ratio; CE, cost-effectiveness; NMB, net monetary benefit. We report confidence intervals for the point estimate of net benefit, but not for the ICER. Confidence intervals for the ICER can be both difficult to interpret^34^ and statistically intractable.^33^ Instead, we place an emphasis throughout our analysis on net benefit. We present cost-effectiveness acceptability curves and confidence intervals around net benefit to represent uncertainty in our cost-effectiveness results.

**Fig. 1:**
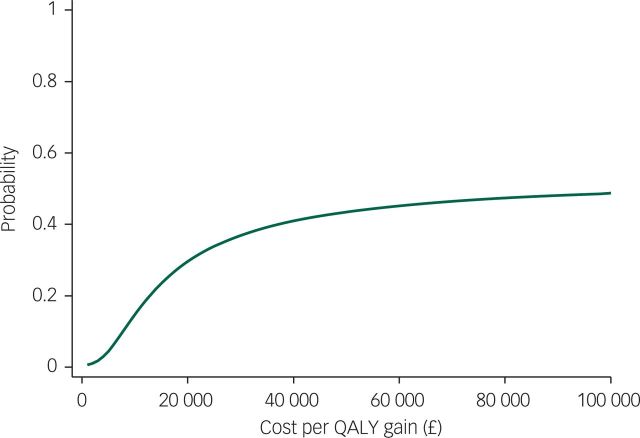
Cost-effectiveness acceptability curve from an NHS/PSS perspective for imputed model.

The QALY difference between arms is small (1/1000th of a QALY), equivalent to less than half a day in perfect health, and is associated with incremental costs of £168. Figure 1 indicates the probability that the intervention is cost-effective at different values of the cost-effectiveness threshold. The intervention is not cost-effective at the NICE thresholds of either £20 000 or £30 000.

### Sensitivity analyses

The results are not sensitive to the exclusion of secondary NHS costs. The effect of removing these costs is to slightly narrow the difference in cost between arms relative to the base case, but to leave the modest between-arm QALY difference unchanged. The intervention was not cost-effective under this scenario (Table 6).

**Table 6:** Sensitivity analysis: cost-effectiveness of the intervention from an NHS/PSS perspective, excluding non-primary-care-/non-intervention-related costs, on imputed data

	Usual care mean	Intervention mean	Difference (95% CI)
Costs and QALYs			
Total NHS/PSS costs	£488	£623	£135 (£70 to £199)
QALYs	0.540	0.541	0.001 (−0.023 to 0.026)
Cost-effectiveness statistics			
ICER: £114 624			
Probability that intervention cost-effective at CE threshold of £20 000: 0.33			
Probability that intervention cost-effective at CE threshold of £30 000: 0.40			
NMB at threshold of £20 000 (95% confidence interval): −£111 (−£132 to −91)			

CI, confidence interval; PSS, personal social services; QALYs, quality-adjusted life years; ICER, incremental cost-effectiveness ratio; CE, cost-effectiveness; NMB, net monetary benefit. We report confidence intervals for the point estimate of net benefit, but not for the ICER. Confidence intervals for the ICER can be both difficult to interpret^34^ and statistically intractable.^33^ Instead, we place an emphasis throughout our analysis on net benefit. We present cost-effectiveness acceptability curves and confidence intervals around net benefit to represent uncertainty in our cost-effectiveness results.

Two further deterministic sensitivity analyses were conducted – one in which the cost of BWW licences was reduced by 50% and another in which the cost of these licences was set to zero. These changes had very minor effects on estimated net benefit and in both cases the intervention was not cost-effective (results not reported).

The cost-effectiveness analysis was re-run on complete cases from an NHS/PSS perspective. In contrast to the analysis on imputed data, this analysis suggested that the intervention is cost-effective with a high probability (Table 7). The CEAC for the complete case analysis is presented in the online supplementary material (Fig. DS2).

**Table 7:** Cost-effectiveness complete case from an NHS/PSS perspective in the depression trial

	Usual care mean (*n*=155)	Intervention mean (*n*=144)	Difference (95% CI)
Costs and QALYs			
Total NHS/PSS costs – complete case	£719	£864	£145 (−£11 to £300)
QALYs – complete case	0.535	0.573	0.037 (0.009 to 0.066)
Cost-effectiveness statistics			
ICER: £3850			
Probability that intervention cost-effective at CE threshold of £20 000: 0.98			
Probability that intervention cost-effective at CE threshold of £30 000: 0.99			
NMB at threshold of 20 000 (95% Confidence interval): £607 (£572 to 642)			

CI, confidence interval; QALYs, quality-adjusted life years; ICER, incremental cost-effectiveness ratio; CE, cost-effectiveness; NMB, net monetary benefit. We report confidence intervals for the point estimate of net benefit, but not for the ICER. Confidence intervals for the ICER can be both difficult to interpret^34^ and statistically intractable.^33^ Instead, we place an emphasis throughout our analysis on net benefit. We present cost-effectiveness acceptability curves and confidence intervals around net benefit to represent uncertainty in our cost-effectiveness results.

## Discussion

Analysis of the primary clinical endpoint indicated that the intervention was effective when measured by the pre-specified primary outcome of proportion of responders in each arm measured by PHQ-9 scores at 4 months.^14^ This improvement is less evident in the 12-month between-arm QALY comparisons and therefore in the overall cost-effectiveness analyses. However, any comparison between the primary clinical outcome and the cost-effectiveness outcome must account for the differences between the outcomes (proportion of responders measured using PHQ-9 and cost-effectiveness using NHS/PSS costs and QALYs), the different time periods over which the outcome is measured (at 4 months and over 12 months) and the overall effect size.

Three considerations are important in interpreting the small between-arm QALY difference. The first is that these differences are consistent with the modest mean effect size in analysis of the intervention on the primary PHQ-9 clinical outcome and the possibility that the intervention was helpful for some but not all participants.^14^ Analysis of secondary trial outcomes indicated greater improvements in anxiety, and greater satisfaction with support, among those randomised to the intervention.^14^

The second consideration is that analysis of correlation (using Spearman's rho) indicates that high PHQ-9 scores (indicating greater severity of depression) and low EQ-5D scores (indicating lower quality of life) are correlated at baseline and all follow-up time points. The null of independence was rejected in each case (*P*<0.001), and the absolute value of the correlations were largest with the anxiety/depression domain of the EQ-5D instrument. This is tentative evidence that the two instruments indicate the same ‘direction of travel' for outcomes, and that the lower sensitivity^21^ of the EQ-5D instrument used in the QALY calculations is not as important to the analysis as the finding that the intervention exerted a positive but modest impact on depression and particularly on health-related quality of life.

The third consideration relates to the amount of missing data. Some 51% of participants had incomplete data on variables necessary to conduct an inferential cost-effectiveness analysis. This was in spite of near-complete data on primary care resource use, prescriptions and intervention cost. Incomplete questionnaire responses to questions on quality of life and NHS resource use account for almost all of the missing data. This was largely because of licencing restrictions imposed on the use of the EQ-5D questionnaire, which had to be sent separately from the main follow-up questionnaires, and had to be sent by post when most outcomes were collected online.

The incremental costs of the intervention arm did not change drastically between imputed cases, available cases and complete cases, largely because of near-complete data on the intervention (the major element of intervention arm costs) and on primary care data. The estimated QALYs from the imputed dataset, adjusting for baseline differences, differed by 0.001 between groups, compared with 0.037 QALYs in complete cases. Below, we explain why the complete case analysis is likely to be both biased and inefficient.

### Strengths

The Healthlines RCT was designed as a pragmatic, theory-based intervention to support patients with a prevalent long-term condition. To our knowledge, it is one of the largest RCTs conducted of a complex intervention based on telehealth for patients with depression. The design and implementation of the intervention itself was the evidence-based culmination of a broad body of work intended to support the development of a responsive, flexible telehealth service.

This economic evaluation adds to the limited evidence base on the cost-effectiveness of telehealth^37^ and internet interventions for mental health.^38^ The economic evaluation was based on analysis of extensive and detailed patient-level data and, unlike a number of studies included in the systematic review of Mistry *et al*,^37^ was conducted with reference to guidelines for best practice in economic evaluation.^39^

### Limitations

The amount of missing cost and quality of life data poses challenges for the analysis and interpretation of results. We attempted to moderate any undue influence of the specific imputation model implemented by following recommended practice in multiple imputation. We compared results of available, complete and imputed cases to assess the reasonableness of the outputs from the imputation model.

It is notable that there was a marked difference between the results using imputed data or complete cases. Estimated NHS costs are similar between complete, imputed and available cases. The pattern of missing EQ-5D data between different cases is summarised in Table 8.

**Table 8:** Quality of life (EQ-5D) data in available, complete and non-complete cases

Quality of life	Usual care		Intervention	
	Mean	*N*	Mean	*N*
Baseline				
All available data	0.52	268	0.51	273
Complete cases	0.52	155	0.54	144
Non-complete cases^a^	0.53	113	0.49	129
4-month follow-up				
All available data	0.53	233	0.56	220
Complete cases	0.52	155	0.59	144
Non-complete cases^a^	0.56	78	0.50	76
8-month follow-up				
All available data	0.54	227	0.56	210
Complete cases	0.53	155	0.59	144
Non-complete cases^a^	0.57	72	0.48	66
12-month follow-up				
All available data	0.57	225	0.57	218
Complete cases	0.56	155	0.58	144
Non-complete cases^a^	0.60	70	0.56	74

a Non-complete cases in this table refer to participants who did not have complete EQ-5D data at all time points. The number of available observations at each time point is therefore the sum of complete and non-complete cases.

Complete case analysis is likely to be inefficient because it would discard data from half of all trial participants. This analysis would be biased because missingness in quality of life data seems to be conditional on observed allocation to the intervention arm, since participants in the intervention who did not provide data at all time points had, on average, lower quality of life than those who provided complete data (Table 8). In the control group, the reverse is true: non-responders to the questionnaires had, on average, a higher quality of life than the complete cases. Table 8 suggests a mechanism for the complete and imputed QALYs to differ.

The amount of missingness in quality of life data complicates comparisons with other trials. For example, the economic evaluation^40^ of the CADET primary care trial of collaborative care,^41^ involving structured and scheduled patient follow-ups and enhanced communication between medical professionals, determined that the intervention was likely to be cost-effective. An important difference between the studies is the amount of missing data in each trial, with CADET reporting missingness of up to 25% compared with 51% in Healthlines.

The level of adherence to the intervention is another potential limitation. The median number of telephone encounters known to have been initiated was 5, out of a total of 10 scheduled encounters, although a focus on the number of completed telephone encounters does not reflect all aspects of compliance with the intervention. There is a lack of methodological guidance concerning how adherence should be reflected in economic evaluation,^42^ albeit the focus on actual adherence in the base-case analysis reflects the pragmatic design of the trial. A complier-average causal effect analysis, using the principal stratification method, of the main trial results^13^ suggested greater effectiveness among participants who received more telephone encounters, although this did not account for baseline variables.

Other limitations include the trial follow-up period, which was limited to 12 months. Longer-term follow-up would be necessary to establish persistence of effect and whether ongoing versus time-limited telehealth support would be most appropriate for this patient group.

It is not clear how closely the operation of the Healthlines service would reflect a system-wide implementation. In practice, scale effects and alternative rostering of HIAs and scheduling of calls may secure more efficient operation, although there is no evidence from the trial itself to indicate that substantial efficiency improvements were available but left unexploited.

In order for the intervention to be cost-effective, it is likely that it would need to be more effective rather than less costly. Holding incremental costs constant, and ignoring the effects of uncertainty, the threshold incremental QALY difference necessary to result in an ICER<£20 000 is approximately 0.085 or about eight times the effect size actually observed in the trial. Improving the effectiveness of the intervention is likely to require better targeting of the intervention to those interested in using it, efforts to improve patient engagement and more effective optimisation of anti-depressant medication when patients fail to respond to treatment.^14^

In conclusion, the Healthlines service was found to be acceptable to patients as a means of condition management,^18^ and response to treatment after 4 months was higher for participants randomised to the intervention.^14^ However, the intervention was associated with a small incremental QALY gain at 12 months and was not likely to be cost-effective at a threshold value of £20 000.
